# Funneled Landscape Leads to Robustness of Cell Networks: Yeast Cell Cycle

**DOI:** 10.1371/journal.pcbi.0020147

**Published:** 2006-11-17

**Authors:** Jin Wang, Bo Huang, Xuefeng Xia, Zhirong Sun

**Affiliations:** 1 Department of Chemistry and Department of Physics, State University of New York at Stony Brook, Stony Brook, New York, United States of America; 2 State Key Laboratory of Electroanalytical Chemistry, Changchun Institute of Applied Chemistry, Chinese Academy of Sciences, Changchun, People's Republic of China; 3 Department of Biological Science and Biotechnology, Ministry of Education Key Laboratory of Bioinformatics, Tsinghua University, Beijing, China, People's Republic of China; University College London, United Kingdom

## Abstract

We uncovered the underlying energy landscape for a cellular network. We discovered that the energy landscape of the yeast cell-cycle network is funneled towards the global minimum (G0/G1 phase) from the experimentally measured or inferred inherent chemical reaction rates. The funneled landscape is quite robust against random perturbations. This naturally explains robustness from a physical point of view. The ratio of slope versus roughness of the landscape becomes a quantitative measure of robustness of the network. The funneled landscape can be seen as a possible realization of the Darwinian principle of natural selection at the cellular network level. It provides an optimal criterion for network connections and design. Our approach is general and can be applied to other cellular networks.

## Synopsis

Cellular networks are in general quite robust and perform their biological functions against environmental perturbations. There are so far very few studies of why networks should be robust and perform biological functions from the physical point of view. In this work, Wang, Huang, Xia, and Sun studied the global properties of the network from physical perspectives. The aim of this paper is to provide a conceptual framework and a tool to study the global nature of the cellular network. The main conclusion is that by uncovering the underlying potential landscape of the budding yeast cell cycle the authors show that it is funneled and robust against the perturbation from kinetic rates and environmental disturbances through noise. This provides the physical explanation of the robustness and stability of the network for performing biological functions. They believe the energy landscape is useful in exploring global properties of protein–protein interaction networks. They also believe the funneled landscape may provide a possible quantitative realization of the Darwinian principle of natural selection at the cellular network level. Finally, Wang et al. derived a quantitative criterion for robustness of the network function. This criterion may provide a novel algorithm for optimizing the network connections to improve the design of synthetic networks.

## Introduction

In the “post-genome” era, it is crucial to uncover the underlying mechanism of cellular networks to understand their biological function [1–3]. The underlying nature of cellular networks has been explored by genetic techniques [4]. Cellular networks have been found to be generally quite robust and to perform their biological functions against environmental perturbations. There are increasing numbers of studies on the global topological structures of networks recently [5] in which the scale-free properties and hierarchical architectures for networks have been found [6–8]. The hubs, highly connected nodes in the network essential for keeping the network together, might play an important role for the robustness of the network. However, there are so far very few studies of why the network should be robust and perform the biological function from the physical point of view [9–18].

Theoretical models of cellular networks have often been formulated with a set of chemical rate equations. These dynamical descriptions are inherently local. To probe the global properties, one often has to change the parameters. The parameter space is huge. The global robustness therefore is hard to see from this approach.

Here we will explore the nature of networks from another angle and formulate the problem in terms of a potential function or potential energy landscape. If the potential energy landscape of the cellular network is known, the global properties can be explored [19,20]. This is in analogy with the fact that the global thermodynamic properties can be explored when knowing the inherent interaction potentials in the system. For the set of the normal chemical rate equations describing the cellular networks, 𝑥̇ *= F*(*x*) with *x* being the concentrations of proteins and *F* being the chemical reaction rate flux (see details in the Methods section), one cannot in general write the right-hand side of these equations as the gradient of a potential energy function. However, typical chemical reaction network equations are only approximations on the average concentration level. In the cell, statistical fluctuations coming from the finite number of molecules (typically on the order of 1–1,000) provide the source of intrinsic internal noise, and the fluctuations from highly dynamical and inhomogeneous environments of the interior of the cell provide the source of the external noise for the networks [21–26]. Both the internal and external noise play important roles in determining the properties of the network.

In general, one should study the chemical reaction network equations in noisy conditions to model cellular environments more realistically. One can also study steady-state properties of these chemical reaction equation networks under noisy environments. The generalized potential for the steady state of the network exists in general [13,15–18,27]. Once the network problem is formulated in terms of the generalized potential energy function or potential energy landscape, the issue of the global stability or robustness is much easier to address. In fact, it is the purpose of this paper to study the global robustness problem directly from the properties of the potential landscape of the network.

To explore the nature of the underlying potential landscape of the cellular networks, we will study the yeast cell-cycle network. One of the most important functions of the cell is the reproduction and growth. It is therefore crucial to understand the cell cycle and its underlying process. The cell cycles during development are usually divided into several phases: the G0/G1, S, G2, and M phases. In most eukaryotic cells, the elaborate control mechanisms over DNA synthesis and mitosis make sure that the crucial events in the cell cycle are carried out properly and precisely. Physiologically, there are usually three checkpoints (where cells are in the quiescent phase waiting for the signal and suitable conditions for further progress in the cell cycle) for controlling and coordination: G0/G1 before the new round of division, G2 before the mitotic process begins, and M before segregation.

Recently, many of the underlying controlling mechanisms are revealed by genetic techniques such as mutations and gene knockouts. It has been found that control has been centered around cyclin-dependent protein kinases (CDKs), which trigger the major events of the eukaryotic cell cycle. For example, the activation of the cyclin/CDK dimer drives the cells at both the G1 and G2 checkpoints for further progress. During other phases and checkpoints CDK/cyclin are activated. Although molecular interactions regulating the CDK activities are known, the mechanisms of the checkpoint controls are still uncertain [9–12].

In Figure 1, a coarse-grained relationship between cyclin and cdc2 in the cell cycle is illustrated. In step 1, cyclin is synthesized de novo. Newly synthesized cyclin may be unstable (step 2). Cyclin combines with cdc2-P (step 3) to form “pre-MPF.” At some point after heterodimer formation, the cyclin subunit is phosphorylated. The cdc2 subunit is then dephosphorylated (step 4) to form “active MPF.” In principle, the activation of MPF may be opposed by a protein kinase (step 5). Nuclear division is triggered when a sufficient quantity of MPF has been activated, but concurrently active MPF is destroyed in step 6. Breakdown of the MPF complex releases phosphorylated cyclin, which is subject to rapid proteolysis (step 7). Finally, the cdc2 subunit is phosphorylated (step 8, possibly reversed by step 9), and the cycle repeats itself.

**Figure 1:**
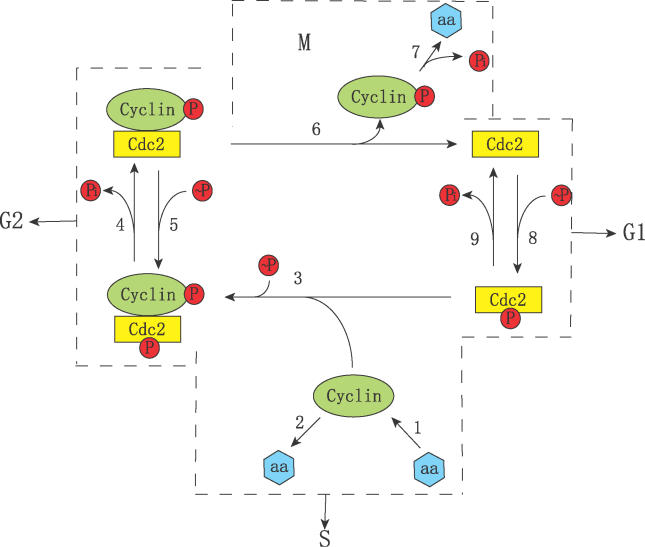
Cell Cycle Network Scheme aa, amino acids; ∼P, ATP; Pi, inorganic phosphate

Mathematical models of the cell cycle controls have been formulated with a set of ordinary first-order (in time) differential equations (chemical rate equations) mimicking the underlying biochemical processes [9–12,14]. The models have been applied to the budding yeast cycle and have explained many qualitative physiological behaviors. The checkpoints can be viewed as fixed points. Since the intracellular and intercellular signal transduction induces the changes in the regulatory networks, the cell cycle can be described by or mimicked by the dynamics in and out of the fixed points. Although detailed simulations give some insights towards the issues, due to the limitation of the parameter space search it is difficult to perceive the global or universal properties of the cycle networks (for example, for different species). It is the purpose of the current study to address this issue.

We will develop a global energy landscape theory for the cell cycle network. This statistical-based approach is good for two reasons. It is a coarse-grained approach that captures only the most important factors, so that the analysis can be carried out relatively easily, revealing some global properties. On the other hand, the statistical approach can be very useful and informative when the data are rapidly accumulating. In this picture, there are many possible states of the network corresponding to different patterns of activation and inhibition of the protein states. Each checkpoint can be viewed as a basin of attractions of globally low energy states. The G0/G1 phase states should have the lowest global energy since it is the end of the cycle. To initiate the new cycle, the network has to receive the signal to activate or pump to the next phase to proceed. The dynamics of the cell cycle are described as the dynamical motions on the landscape state space from one basin to another. This kinetic search is not entirely random but directed, since the random search takes cosmological time. The direction or gradient of the landscape is provided from the tilting towards the G0/G1 phase. The landscape therefore becomes funneled towards the G0/G1 state, with the bottom of the funnel what we call the native state. At the end of G0/G1 phase, the network is pumped to high energy excited states at the top of the funnel (cycling). The cell cycle then follows as it cascades through the configurational state space (or energy landscape) in a directed way, passing several checkpoints (basins of attraction), and finally reaching the bottom of the funnel–G0/G1 phase again before being pumped again for another cycle. We will study the global stability by exploring the underlying potential landscape for the yeast cell-cycle network.

The aim of this paper is to provide a framework and a tool (potential energy function) to study at the cell network globally. At the conclusion of this paper we show that the potential landscape of the budding yeast cell cycle is funneled and robust against the perturbation from the kinetic rates and the environmental disturbances through noise.

## Methods

### Average Kinetics

Here, we start with a quantitative computational description of the relationship between cyclin and cdc2 and the associated chemical reaction rate processes in the yeast cell cycle. See Figure 1.

Based on the law of mass action, one can derive a set of differential equations that describe the variation rate of each component's concentration for each component of the relations above—the chemical reaction rate equations. Together with the conservation equations, we have five independent simplified equations (Equations F1–F5 are components of chemical reaction rate flux). The rate constants are experimentally measured or inferred [9–12]:

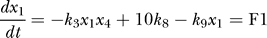


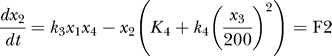


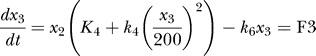


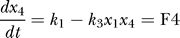


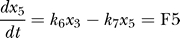


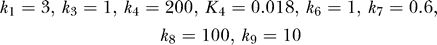


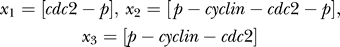


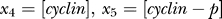


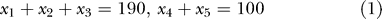


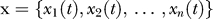
are the concentrations of the different proteins in the network. *F*(*x*) is the “force or chemical reaction rate flux term” involving the chemical reactions which are often nonlinear in protein concentrations *x* (for example, enzymatic reactions as shown above). The *k*'s are the kinetic rate coefficients. The 𝑥̇ *= F*(*x*) equation describes the averaged dynamical evolution of the chemical reaction network in the bulk.

### Potential Landscape from Noisy Environments

Due to the intrinsic statistical fluctuations of the protein (kinase) numbers in the limited cell volume and external fluctuations within cellular environments, the described averaged chemical-rate equations above cannot faithfully describe the inherent process and need to be modified. The statistical fluctuations can be very significant from both internal and external sources and in general cannot be ignored [21–26]. A stochastic force *ζ* can then be added as the noise mimicking these fluctuations. The distribution function of the noise is assumed to be Gaussian, from the large number theorem in statistics. It is equivalent that the mean of the noise terms are zero: <*ξ_i_*(*t*) > = 0. Then the auto correlations of the noise are given by:

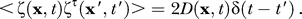



Here *δ*(*t*) is the Dirac delta function, and the diffusion matrix *D* is explicitly defined by < *ζ_I_* (*t*)*ζ_j_* (*t*′) > = 2*D_ij_δ* (*t–t*′). The average <…> is carried out with the Gaussian distribution for the noise.

We add noise sources to each rate equation and derive five stochastic differential equations:

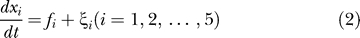



The stochastic trajectories of each individual variable (in this case each concentration of proteins) satisfying the equation of motion with noise are not deterministic. Therefore they are better characterized by the statistical distributions of the inherent variables (protein concentrations, in this case) [13,15–18,28]. In fact, the multidimensional stochastic equation of motion is equivalently described by the corresponding time evolution of the statistical distribution satisfying the multidimensional Fokker-Planck equation in macroscopic conditions [27,13,28]. By taking the long time limit, the steady-state distribution in concentration space can be obtained.

We can naturally define a generalized potential function *U* based on the steady-state distribution function in multidimensional concentration space as *P_steady–state_* ∼ *exp*[–*U*] or *U* ∼ –*ln*(*P*_steady–state_). In this definition of potential *U,* we can see that when a multidimensional concentration configuration has a higher probability of appearing, then the corresponding generalized potential is lower. The maximum of the steady-state probability *P_steady–state_* corresponds to the minimum of potential energy *U*. Knowing the steady-state distribution, one can map out the corresponding potential energy landscape. There is a one-to-one correspondence between the steady-state probability and the generalized potential [13,15–18,28]. Thus, the issue of finding the generalized potential to explore the global properties becomes an issue of finding the steady-state probability distribution function itself. In principle, we can solve the steady-state probability of the corresponding multidimensional Fokker-Planck equation. In practice, this is quite a challenging mathematical task.

Instead of directly solving the multidimensional Fokker-Planck equation, we will follow a scheme of directly constructing the potential U from the stochastic equation of motion through a transformation without the need to solve the corresponding Fokker-Planck equation [15]. We will in the rest of the text make the assumption of the transformation first and then show that this particular mathematical construction of the potential U directly relates to the steady-state probability from the Fokker- Planck equation through *P_steady–state_* ∼ *exp*[–*U*]. Furthermore, we will use this scheme of mathematical construction to directly obtain the generalized potential *U*.

Let us assume a transformation [15], such that we can write the network equations in the following form:

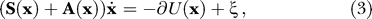
with the semipositive definite symmetric matrix function **S**(**x**), the antisymmetric matrix **A**(**x**), the single-valued scalar function *U*(**x**), and the stochastic force *ξ*. Here ∂ is the gradient operator in the state variable space. It is important to realize that the semipositive definite symmetric matrix term is “dissipative”: 𝑥̇^*T*^ *S*(x) 𝑥̇ > = 0 (**x** *^T^* is the transpose vector of **x**). The antisymmetric part does no “work”: 𝑥̇^*T*^ *A*(**x**) 𝑥̇ = 0, and therefore is nondissipative. Hence, it is natural to identify that the dissipation is represented by the semipositive definite symmetric matrix **S**(**x**), the friction matrix, and the transverse force by the antisymmetric matrix **A**(**x**), the transverse matrix. In general, we cannot write F(x) as a gradient of a potential energy function, and therefore no potential energy can be defined in noise-free environments. However, through the transformation, there exists a gradient term involving *U* in the presence of noise. The scalar function *U*(**x**) then acquires the meaning of potential energy.

There are four independent measures **S**,**A**,**U**,*ξ* in the above equation while there are only two **F**,*ζ* in equation d**x**/dt** = F +** *ζ*. Additional constraints need to be imposed to obtain a unique solution for the above equation. To be consistent with the Gaussian and white noise assumption for *ζ,* we may naturally impose the constraint on the stochastic force and semipositive definite symmetric matrix to be [15]:

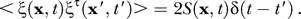



The above relationship resembles the dissipative dynamics of quantum mechanics when both the dissipative force and Berry phase exist. It relates the stochasticity with the dissipation or the fluctuation–dissipation theorem of the second kind [29].

To illustrate this construction of the potential, we can use the equation d**x**/dt** = F +** *ζ* to eliminate the 𝑥̇ in equation (S(x)+A(x)) 𝑥̇ = –∂*U*(x)+ ξ, yielding [15]:

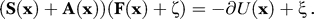



Notice that the dynamics of the noise should be independent of that of the deterministic dynamics. Thus we require both the deterministic force and the noise to satisfy two separate equations. For the deterministic force, this reads:

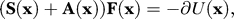
and for the noise term, we have:

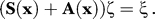



The above transformations effectively “rotate” the deterministic force to the gradient of the potential *U* and stochastic force from *ζ* to *ξ* (with the same rotation) at every point in state space.

Using equation < *ζ*(**x**,*t*)*ζ^τ^*(**x**′,*t*′) > = 2*D*(**x**,*t*)*δ*(*t* – *t*′) and < *ξ*(**x**,*t*) *ξ^τ^*(**x**′,*t*′) > = 2*S*(x,*t*)*δ*(*t* – *t*′), equation (**S**(**x**) + A(**x**))*ζ* = *ξ* becomes:

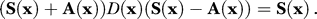



This suggests a duality between the friction matrix S and diffusion matrix D where a large diffusion matrix implies a small friction matrix. It is a generalization of the Einstein fluctuation–dissipation relationship to a nonzero transverse matrix A.

We can introduce the auxiliary matrix function G [15]:

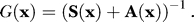



Here “–1” means the inverse of the corresponding matrix. Thus the inverse function of *G* is given as *G* ^−1^(**x**) = **S**(**x**) + A(**x**) and the transpose of inverse function of *G* is given as (*G* ^−1^)*^t^*(**x**) = **S**(**x**) − **A**(**x**). Using the property of the potential *U*: ∂ × ∂*U* = 0, from equation (**S**(**x**) + **A**(**x**))**F**(**x**) = –∂*U*(**x**), we obtain:

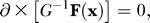
which gives the *n*(*n* – 1)/2 conditions to determine the *n* × *n* auxiliary matrix function *G*. The generalized Einstein relationship (**S**(**x**) + **A**(**x**))*D*(**x**)(**S**(**x**) – **A**(**x**)) = **S**(**x**) combining the relationships of *G* ^−1^(**x**) = (**S**(**x**) + **A**(**x**)) and (*G* ^−1^)*^t^*(**x**) = (**S**(**x**) – **A**(**x**) leads to:

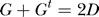



The above equation determines the other *n*(*n* + 1)/2 conditions for *G*. Thus the above two equations completely determine *G*.

It is straightforward to show from equation (**S**(**x**) + **A**(**x**))**F**(**x**) = –∂*U*(**x**), the definition of *G*, and the relationships of *G* ^−1^(**x**) = (**S**(**x**) + **A**(**x**)) and (*G* ^−1^)*^t^*(**x**) = (**S**(**x**) – **A**(**x**)) that:

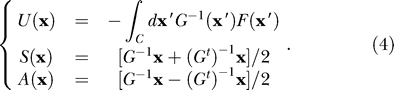



Thus the potential function *U,* the friction matrix *S,* and transverse matrix *A* can be completely solved.

### Potential and Steady-State Probability from the Fokker-Planck Diffusion Equation

We can prove in this section that the potential constructed in the last section is directly linked with the steady-state probability distribution of the corresponding master equation [15]. Since the trajectories are stochastic with the presence of noise, it is more appropriate to describe the system in terms of probability distribution function rather than the individual trajectory or only the average of the trajectories. The connections between the stochastic approach and the probabilistic approach can be established through the connections between stochastic differential equations and diffusional Fokker-Planck equations [27]. The deterministic force and stochastic force are on different time scales. The deterministic time scale is typically larger than the stochastic fluctuation time scale. The separation of time scales suggests that network dynamics have an inertial. We can introduce the inertial mass *m* and kinetic momentum *p* for the network [15]:

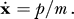



The above equation defines the momentum, and

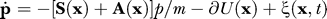
is the extension of equation (**S**(**x**) + A(**x**)) 𝑥̇ = – ∂*U*(**x**) + *ξ* when including the inertial mass. Since there is no dependence of the friction matrix and the stochastic force on the kinetic momentum, there is no Ito-Stratonovich dilemma connecting the stochastic differential equation and the evolution equation of the probability distribution function [27]. The Fokker-Planck equation in this enlarged state space can be written as [15]:

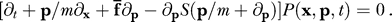



Here f̄ = **p** *A / m –* ∂_x_ *U* and *t*, **x** and p are independent variables of time, space, and momentum. The steady-state distribution function can be found to be:

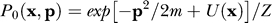
with the partition function Z = *d^n^* **x** *d^n^* **p** *exp*[–**p** ^2^ / 2*m* + *U*(**x**)]. In the above equation, the state variables and kinetic momentum are explicitly separated. The zero mass limit (no inertial) can be taken, which does not cause any effects on the state variable distribution.

The steady-state distribution function *P*_0_(**x**) for the state variable **x** can thus follow a Boltzman-Gibbs distribution, which is exponential in potential energy function *U*(**x**) (after integrating out the momentum variables) [15]:

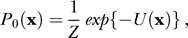
with the partition function Z = *d^d^x* exp{–*U*(**x**)}. From the steady-state distribution function, we can therefore identify *U* as the generalized potential energy function of the network system. In this way, we map out the potential energy landscape. Once we have the potential energy landscape, we can discuss the global stability of the protein cellular networks. Following are the detailed descriptions of the calculation procedures.

### Detailed Calculations of the Potential

The detailed mathematical expression of friction force *S,* transverse force *A,* and potential *U* were given as Equation 4 as mentioned in the above sections [15]:

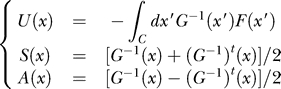
with *G* matrix function and its transpose *G^t^* satisfying constraint equations (*G* ^−1^ is the inverse function of *G*) as mentioned in the above sections:

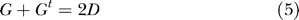
and ∂ × [*G* ^−1^ **F**(**x**)] = 0 leads to:

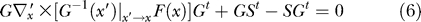



One can approximate the above equation in gradient expansions to zero, first, and higher orders to solve the *G* matrix function and substitute the solution to obtain the potential *U*. For simplicity we only solve matrix *G* and corresponding *U* up to zero and first order. We found convergent solutions.

The zeroth order approximation of *G* is given below:

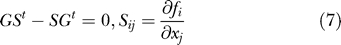



We assume D is a diagonal matrix *D*_0_ *I* (*I* is identity matrix) for simplicity; further, we take *D*_0_ as constant 1.Then for the first-order solution, from Equation 5 and Equation 6, we can solve the linear set of equations for *G*. Taking *G* in Equation 4 and performing the calculation of the integral, we get the potential *U*.

The diffusion coefficient *D* in general might be dependent on the concentration. It is, however, quite difficult to estimate the exact functional form of the diffusion on concentration. If we assume that the diffusion is slowly varying on concentrations, then we can expand the diffusion around a fixed point and extend it to the other regions. As a first approximation, we treated the diffusion here as a constant for simplicity. This should be at the exact fixed point. Since we don't know the diffusion scale exactly, we can set the constant diffusion coefficient *D* to be equal to 1 for simplicity. The scale of protein concentration variation ranges from 0–∼100. *D* = 1 is then significantly smaller than 100 and corresponds to weak noise. For stronger noise (*D* >> 1), the whole network will be destroyed and therefore will not function properly.

### Numerical Solution

Equation 5 is a set of linear equations that can determine 5*(5 + 1)/2 conditions. Equation 6 can determine 5*(5 – 1)/2 conditions.

For the higher-order approximation, we have an iteration equation that takes the zero order result as the initial value; the equation reads:

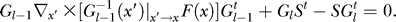



This equation also determines 5*(5 – 1)/2 conditions. After we have *G* and *G* ^−1^, we can easily integrate Equation 4 to obtain the potential *U*.

In the process of solving the linear set of equations for *G,* we first normalized the concentrations and then divided them into 20–1,000 bins. We solved the problem exactly with fewer bins and datapoints, but used the Monte Carlo method to sample the data with more bins and datapoints. We solved *G* up to zero and first order and found convergent solutions.

The protein concentrations of the global minimum of the potential energy landscape are found to be at the G0/G1 native state (x1 = 1, x2 = 1, x3 = 190, x4 = 25, x5 = 80).

## Results/Discussion

The potential energy function *U*(*x*) is directly linked with the probability of the configuration state characterized by protein concentration *x*. Low energy of a particular state corresponds to a high probability of occurrence of the state. Different configurational states of the cellular network therefore have different probabilities of occurring, and therefore different, energies. Since the potential energy is a multidimensional function in concentration configuration space *x,* it is difficult to visualize *U*(*x*). In Figure 2, we do a zero- (Figure 2A) and 1-D (Figure 2D and 2E) projection and look at the nature of the underlying potential energy landscape *U*.

**Figure 2:**
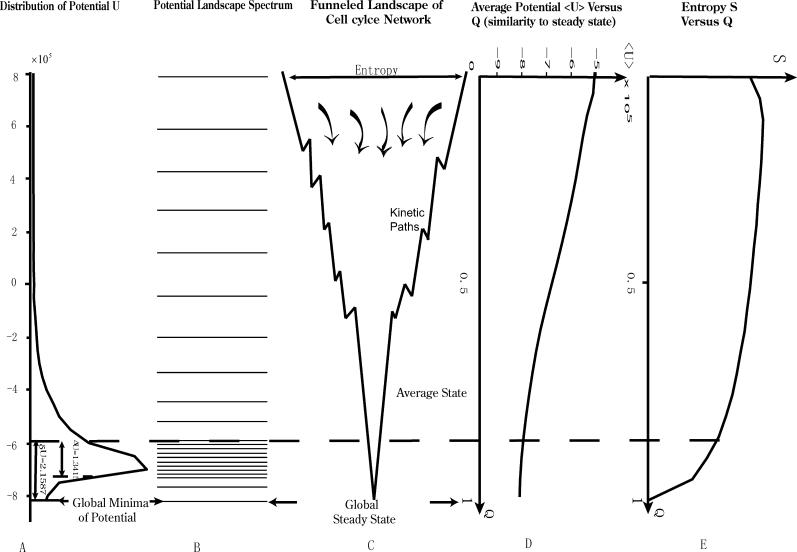
The Global Structures and Properties of the Underlying Potential Landscape of the Yeast Cell–Cycle Network (A) The histogram or the distribution of the potential U. (B) The potential landscape spectrum. (C) The funnelled landscape of the yeast cell–cycle network. (D) The averaged potential as a function of similarity parameter Q with respect to the global minimum G1 state (or global steady state) of potential U. (E) The entropy as a function of similarity order parameter Q with respect to the global minimum G1 state (or global steady state) of the potential U.

We can see that the distribution is approximately Gaussian. The lowest potential *U* is the global minimum of the potential landscape. It is important to notice that this global minimum of *U* is found to be at the same place (in *x*) as the G0/G1 fixed point or G0/G1 phase for the yeast cell cycle. Figure 2B illustrates the potential energy spectrum. It is clear that the global minimum of the potential energy is significantly separated from the rest of the potential spectrum or distribution.

To quantify this, we define the robustness ratio (RR) for the network as the ratio of the gap δ*U,* the difference between this global minimum of the G0/G1 state 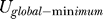 , and the average of *U,* <*U>,*versus the spread or the half-width of the distribution of *U*, Δ*U*, RR = 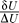 . *δU* is a measure of the bias or the slope towards the global minimum (G0/G1 state) of the potential energy landscape. Δ*U* is a measure of the averaged roughness or the local trapping of the potential landscape. When RR is significantly larger than 1, the gap is significantly larger than the roughness or local trapping of the underlying landscape, and the global minimum (G0/G1 state) is well-separated and distinct from the rest of the cell cycle network potential spectrum. Since *P*_0_(*x*) = 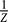 exp{–*U*(*x*)}, the weight or the population of the global minimum (G1 state) will be dominated by the one with large RR. The populations of the rest of the possible configurational states of the cell cycle network are much less significant. This leads to the global stability or robustness discriminating against others. The RR value for the yeast cell–cycle network is RR = 1.61, which is significantly larger than 1. RR thereby gives a quantitative measure of the property of the underlying landscape spectrum. Only the cellular network landscape with a large value of RR will be able to form a stable global minimum G0/G1 state, be robust, perform biological functions, and survive natural evolution.

Figure 2D shows the 1-D projection of the averaged *U*, <*U*>, to the overlapping order parameter *Q* with respect to the global minimum (*Q* = 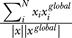 ). *Q* is defined this way so that we can keep track of the degree of “closeness” or overlap between an arbitrary state *x* and the global minimum state *x_global_* in the configurational state space of the protein concentrations. *Q* = 1 represents the global minimum state and *Q* = 0 represents the states with no overlap (decorrelated) with the global minimum. Here the global minimum is at the same place (in *x*) as the G0/G1 phase of the cell cycle. We see a downhill slope of the potential < *U*> in *Q* towards the global minimum *U_global_*. This shows clearly a funnel of < *U*>along *Q* towards the global minimum of the potential landscape. When randomly changing the chemical rate coefficients (10%–20%), the slopes of < *U*> along *Q* towards the global minimum of the potential landscape do not change very much (as shown in Figure 3). So the landscape is still funneled towards the global minimum under different cellular conditions. Therefore the network is relatively stable and robust. With more drastic changes of the rate parameters (above 50%), the landscape starts to become less stable and loses its robustness.

**Figure 3:**
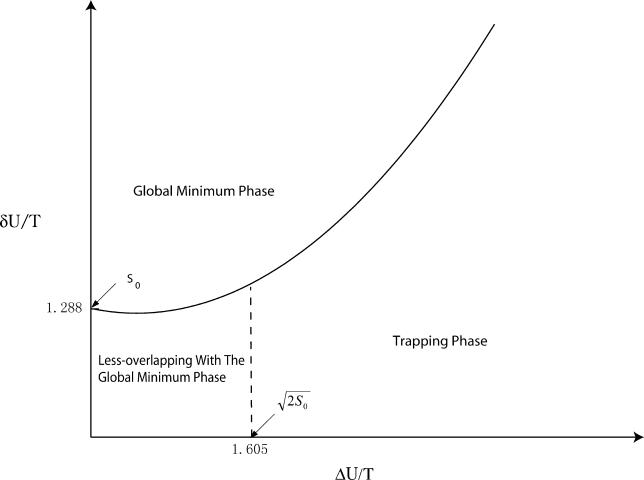
The Averaged Potential U as a Function of Similarity Parameter Q with Respect to the Global Minimum G1 State (or Global Steady State) of Potential U against Perturbations of Chemical Rate Coefficient Parameters with 10% Increase (Decrease), 20% Increase (Decrease)

The system does not have multiple fixed points or multiple energy valleys. In this model, it has only one, which corresponds to the G0/G1 global minimum. In the moderate parameter range we vary as described above, the number of the fixed point or global energy valleys remains one. Therefore, the system is quite robust against perturbations and the underlying energy landscape is funneled towards the G0/G1 global minimum.

Figure 2E shows the configurational entropy *S*_0_ (*Q*) = lnΩ(*Q*); Ω(*Q*) is the number of the configurational states at particular overlap *Q* as a function of *Q*. The entropy is calculated by dividing the concentration variables into a multidimensional lattice, counting the number of states in each multidimensional lattice cube, and then projecting them onto *Q*. As we can see, the entropy is rather smooth at small *Q* and decays as *Q* migrates towards the global steady state or global minimum. Since the entropy represents the number of states available, this implies that the configurational state space for the network becomes smaller towards the global steady state. Thus entropy can be used as a measure of the radius of the funneled landscape perpendicular to the direction of the funnel towards the global steady state (see in Figure 2C that the funnel shrinks in radial size towards the global steady state).

In Figure 4, we construct the free energy versus overlap order parameter *Q*, *F*(*Q*) by making use of the microcanonical ensemble. *F*(*Q*) = *U* – *TS*. *U* and *S* are the potential energy and entropy of the system, respectively. They are given by *U* = < *U* > (*Q*) – 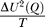 and *S* = *S*_0_ (*Q*) – 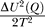 . Here, <*U*>(*Q*) is the average of the potential of *U*(*Q*) at each overlap *Q*; Δ*U* ^2^(*Q*) is the variance of potential at each *Q; S*(*Q*) is the entropy of the configuration at *Q*, and *S*_0_(*Q*) is given by *S*_0_(*Q*) = lnΩ(*Q*); Ω(*Q*) is the number of the configurational states at particular overlap *Q. T* is the effective temperature.

**Figure 4:**
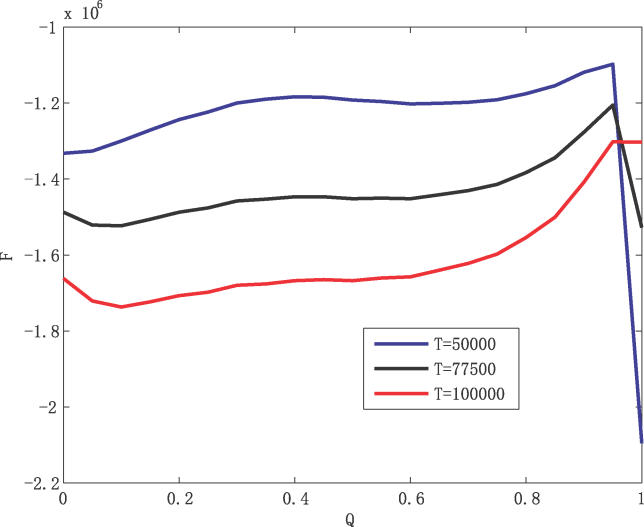
The Free Energy as a Function of Overlap Parameter Q Relative to the Global Minimum G1 Steady-State Fixed Point at Low Temperature (50,000), Intermediate Temperature (77,500), and High Temperature (100,000)

The effective temperature here is a qualitative measure of the influence of the environments to the network, which could be in the form of the stress, radiation, salt changes, pH changes, protein components in the cell cycle interacting with others not in the cell cycle but within the cell, etc. They are qualitatively mimicked by noise here. This is different from protein-folding studies [30]. In protein folding, the temperature is the normal temperature. But here temperature represents qualitatively the strength of the external noise to the system.

The exact meaning and mapping of the noise to the biological process still needs to be worked out. To describe the nature of the statistical fluctuations and the corresponding underlying energy landscape, we consider both strong and weak noise limit, and provide a qualitative analysis here. When the noise is strong or the external influence is strong to the cell cycle system, the system will no longer be robust nor perform biological functions anymore. The corresponding energy landscape becomes less biased towards the biological functioning states. For weak noise, the external influences on the cell cycle network are small; therefore the network should be more robust. This is often true except for the case where the network itself is not very robust, even without external influences. In this limit, the network can get trapped in the local minimum or intermediate states without reaching the destination. This happens if the underlying landscape is rough. To guarantee the robustness of the network, certain noise might be necessary to overcome the barrier to complete the network processes.

In other words, strong noise is bad for stability, which will lead to a “phase transition” from an ordered functional state to the unstructured dis-function state. As mentioned before, here we can use the temperature *T* as a qualitative measure of the strength of the noise. The “temperature” *T*n is the noise level below which the system is stable and functioning. For stability we would like to have this *T*n be high, so that in most situations the system is below this threshold noise level and thus is stable. On the other hand, small noise might not be able to get out of the local traps in order to perform biological functions. There could be a trapping phase transition from an ordered functional state to a trapped state without moving farther. The temperature *Ttrapping* is the noise level below which the system will be trapped and not functioning. For discrimination, we would like to have this *Ttrapping* be low, so that in most situations the system is above this threshold noise level and not trapped. To minimize *Ttrapping* and maximize *Tn* at the same time, the ratio *Tn*/*Ttrapping* should be maximized. We can prove that the requirement that the system is both stable and not trapped can be satisfied by the underlying funneled landscape with a large ratio of the gap biasing towards G1 against the roughness or local trapping depths. Below are the details.

Two characteristic thermodynamic transition temperatures exist in Figure 4. At low temperatures (*T* = 50,000), the free energy is biased towards the global minimum (G1 state) of *U* and the *Q* = 1 state is thermodynamically more stable; at high temperatures (*T* = 100,000), the free energy is biased towards the states that are less correlated with the global minimum of *U*(G1 state), and the *Q* < 1 states are more thermodynamically stable. At intermediate temperature (77,500), most likely around the physiological temperature regime, the expression for free energy can have a double minimum structure on the order parameter *Q*. We can equate the two minima of the free energies from less-overlapping states to the global minimum G1 state of the yeast cell–cycle network to obtain the native transition temperature

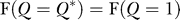


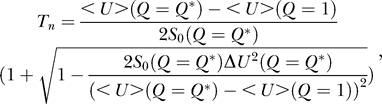
where < *U*>(*Q* = *Q* ^*^) – < *U*>(*Q* = 1) is the gap between the global minimum and the less-overlapped states (the states that have the same free energy as the global minimum G1 state). This is a first-order “phase transition” point, representing the coexistence of the global minimum steady-state phase and another phase with fewer overlaps with the global minimum steady state.

Figure 4 also shows the free energy profile of the network at the native transition temperature (*T* = 775,000). Another possible “phase transition” exists where the entropy of the system goes to zero, which indicates that the system runs out of the states and becomes trapped in the local minimum. This transition gives *S*_0_(*Q* = *Q* ^*^) = 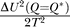 so that the trapping temperature is given by 
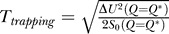
 .

Taking the ratio of native transition temperature to the trapping temperature of the network, we obtain:

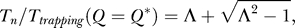
where 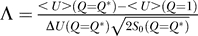 is the ratio of the potential gap between the global minimum state and the average of the potential landscape spectrum versus the ruggedness or the width (spread) of the distribution of the potential landscape spectrum weighted by entropy or the measure of the number of the states available 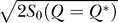 . We can see that the RR is directly related to Λ: 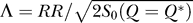 . In other words, Λ is the RR weighted by entropy.

There are at least three possible thermodynamic phases: the global minimum G1 state, the less-overlapping with the global minimum G1 state, and the trapping phase (see Figure 5). The global minimum G1 state in the yeast cell-cycle example corresponds to the final destination at the end of one complete cell cycle. Without further stimulation, the cell will sit at the G1 state and not go into the next stage of development. Clearly, the native transition (to the global minimum G1 state) temperature should be higher than the trapping temperature to guarantee the global thermodynamic stability and avoid nondiscrimination with traps. The ratio*T_n_ / T_trapping_* should therefore be maximized. From the above expression, this is the equivalent of saying that Λ, or RR, should also be maximized. Therefore, maximizing the ratio of the potential gap (or the slope) versus the roughness of the underlying potential landscape weighted by the entropy of the available states (a measure of the configurational search space) becomes the criterion for the global thermodynamic stability or robustness of the network. Only the cellular network landscape satisfying this criterion will be able to form a thermodynamically stable global steady state, be robust, perform the biological functions, and furthermore survive natural evolution. Similar to the problems of protein folding and binding [30,31], this implies a funneled potential landscape of the cellular network as shown in Figure 2C, which has a directed downhill slope biased towards the global minimum G1 state, dominating the fluctuations or wiggles superimposed on the landscape and the configurational search space. From this picture, at the initial stage of the yeast cell-cycle network process, there are multiple parallel paths leading towards the global minimum G1 state. As the kinetic process progresses, the discrete paths might emerge and give dominant contributions when the roughness of the underlying landscape becomes significant.

**Figure 5:**
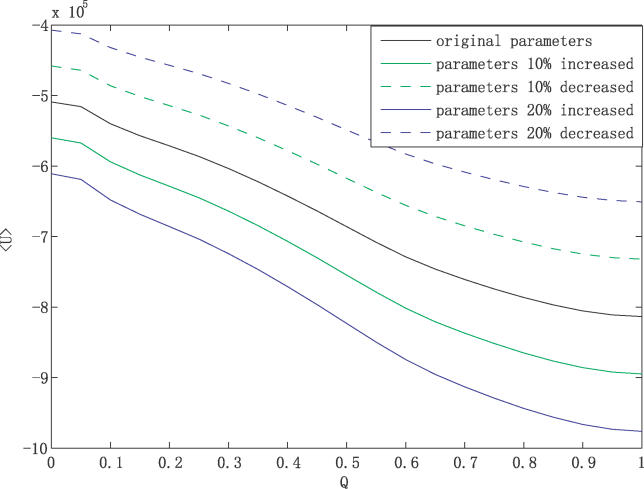
Thermodynamic Phase Diagram for the Yeast Cell–Cycle Network Native phase with global minimum G0/G1 state or steady state; non-native phase with states less overlapping with global minimum G0/G1 state or steady state; trapping phase with states trapped into the local minimum. The larger of δU/T and smaller of ΔU/T, or the larger *δ*U/ΔU, the more likely the global minimum G1 state is thermodynamically stable and robust.

In cell-cycle biology, the G1 (or more accurately G0) phase is the phase in Figure 1 before the first step of synthesis of cyclins (bottom of Figure 1). It is known from the experiments that, without the nutrition and rich supply of amino acids needed, the cyclins do not accumulate and the cell cycle cannot proceed. G0/G1 is the starting point and the end point of the cell cycle. Only upon activation through rich nutrition will the cell start to grow. Without the activation through nutrition, the cell will always sit at the G0/G1 phase. In the landscape picture, G0/G1 is at the bottom of the funnel. Therefore the system at G0/G1 is very stable without the perturbation or activation. On the other hand, with rich nutrition, the cell is activated and proceeds to the other states to complete the cell cycle. In energy landscape language, upon activation, the system is “activated” or “pumped” from the G0/G1 state to the other excited states of the cell so that the consequent dynamics of the downhill motion through the funnel leads to G0/G1 again. The cell cycle begins again only when the system reaches G0/G1 and there is a rich supply of nutrition leading to activation. Therefore the robustness of G0/G1 is critical for the whole cell cycle process. If the G0/G1 phase is not significantly changed, the system is likely to be normal. On the other hand, if G0/G1 is significantly changed upon environmental or internal perturbations, the system is likely to be disturbed and not function properly. The other phases of the cell cycle are likely to be the intermediate metastable states in the landscape picture.

When we explore further the influence of the environmental changes on the other phases of the cell cycle, we reach similar conclusions; for example, the M phase is quite robust against perturbations. The ratio of the underlying energy gap versus roughness is a global quantified measure of the robustness. The perturbation that destroys the stability of G0/G1 will significantly influence the other phases, and therefore the whole system, too. A large RR will guarantee not only the stability of G0/G1, but also other phases of the cycle.

### Conclusions

The cellular network with a rough underlying potential landscape can neither guarantee the global robustness nor perform a specific biological function. They are less likely to be selected in the evolution process. The funneled landscape therefore might provide a possible quantitative realization of the Darwinian principle of natural selection at the cellular network level. As we see, the funneled landscape provides an optimal criterion to select the suitable parameter subspace of cellular networks, guarantees the robustness, and performs specific biological functions. This might lead to optimal network connections. The details of the application of this novel algorithm to improve the design of the synthetic network will be given in a future work. It is worth pointing out that the approach described here is general and can be applied to many cellular networks, such as signaling transduction networks [2], metabolic networks [32], and gene regulatory networks [13,16]. A highly simplified model for lambda phage [15] was studied where only two protein concentration variables were included. It is a much simpler system to study mathematically, and it can be solved exactly. The cell cycle is a much more complicated system that involves many proteins. Even in the most simplified form we consider here, it still has several protein concentration variables to be considered. Therefore, we used a numerical approximation scheme to solve the problem rather than an exact method, which can then be used to treat networks with more than two components.

We also worked on a different model for MAP Kinase signal transduction network [28]. We found that the underlying potential energy landscape is also a funnel, and is robust against rate parameter and external noise perturbations. This leads us to believe the funneled landscape and its robustness might be general. We are now studying the metabolic networks. The results will be published elsewhere.

There are other cell cycle network models (for different species of yeast) that involve more protein concentration variables than the simple one we have here. Since the degrees of freedom grow exponentially with the size of the system, it is difficult to explore larger systems. We are developing approximation schemes now to overcome the computational bottleneck for obtaining the landscape for larger cell network systems.

## Abbreviations

CDK: cyclin-dependent protein kinases
RR: robustness ratio

## References

[pcbi-0020147-b001] Huang Davidson EH (2002). A genomic regulatory network for development. Science.

[pcbi-0020147-b002] Huang CY, Ferrell JE (1996). Ultra sensitivity in the mitogen-activated protein kinase cascade. Proc Natl Acad Sci U S A.

[pcbi-0020147-b003] Kholodenko BN (2000). Negative feedback and ultrasensitivity can bring about oscillations in the mitogen-activated protein kinase cascades. Eur J Biochem.

[pcbi-0020147-b004] Ideker T (2001). Integrated genomic and proteomic analyses of a systematically perturbed metabolic network. Science.

[pcbi-0020147-b005] Jeong H, Tombor B, Albert R, Oltvai ZN, Barabasi AL (2000). The large-scale organization of metabolic networks. Nature.

[pcbi-0020147-b006] Barabasi AL, Bonabeau E (2003). Scale-free networks. Sci Am.

[pcbi-0020147-b007] Maslov S, Sneppen K (2002). Specificity and stability in topology of protein networks. Science.

[pcbi-0020147-b008] Milo R, Shen-Orr S, Itzkovitz S, Kashtan N, Chklovskii D (2002). Network motifs: Simple building blocks of complex networks. Science.

[pcbi-0020147-b009] Tyson JJ, Chen K, Novak B (2001). Network dynamics and cell physiology. Nat Rev Mol Cell Biol.

[pcbi-0020147-b010] Novak B, Tyson JJ (1997). Modeling the control of DNA replication in fission yeast. Proc Natl Acad Sci U S A.

[pcbi-0020147-b011] Tyson JJ (1991). Modeling the cell division cycle: cdc2 and cyclin interactions. Proc Natl Acad Sci U S A.

[pcbi-0020147-b012] Chen KC, Calzone L, Csikasz-Nag A, Cross FR, Novak B (2004). Integrative analysis of cell cycle control in budding yeast. Mol Biol Cell.

[pcbi-0020147-b013] Sasai M, Wolynes PG (2003). Stochastic gene expression as a many body problem. Proc Natl Acad Sci U S A.

[pcbi-0020147-b014] Li F, Long T, Lu Y, Ouyang Q, Tang C (2004). The yeast cell-cycle network is robustly designed. Proc Natl Acad Sci U S A.

[pcbi-0020147-b015] Ao P (2004). Potential in stochastic differential equations: Novel construction. J Phys A Math Gen.

[pcbi-0020147-b016] Zhu XM, Lan L, Hood L, Ao P (2004). Calculating biological behaviors of epigenetic states in the phage lambda life cycle. Funct Integr Genomics.

[pcbi-0020147-b017] Qian H, Bear DA (2005). Thermodynamics of stoichiometric biochemical networks far from equilibrium. Biophys Chem.

[pcbi-0020147-b018] Qian H, Reluga TC (2005). Nonequilibrium thermodynamics and nonlinear kinetics in a cellular signaling switch. Phys Rev Lett.

[pcbi-0020147-b019] Austin RH, Beeson K, Eisenstein L, Frauenfelder H, Gunsalus I (1975). Dynamics of ligand binding to myoglobin. Biochemistry.

[pcbi-0020147-b020] Frauenfelder H, Sligar SG, Wolynes PG (1991). The energy landscapes and motions of proteins. Science.

[pcbi-0020147-b021] McAdams HH, Arkin A (1997). Stochastic mechanisms in gene expression. Proc Natl Acad Sci U S A.

[pcbi-0020147-b022] Elowitz MB, Leibler S (2000). A synthetic oscillatory network of transcriptional regulators. Nature.

[pcbi-0020147-b023] Swai PS, Elowitz MB, Siggia ED (2002). Intrinsic and extrinsic contributions to stochasticity in gene expression. Proc Natl Acad Sci U S A.

[pcbi-0020147-b024] Thattai M, van Oudenaarden A (2001). Intrinsic noise in gene regulatory networks. Proc Natl Acad Sci U S A.

[pcbi-0020147-b025] Vilar JMG, Guet CC, Leibler S (2003). Modeling network dynamics: The lac operon, a case study. J Cell Biol.

[pcbi-0020147-b026] Paulsson J (2004). Summing up the noise in gene networks. Nature.

[pcbi-0020147-b027] Van Kampen NG (1992). Stochastic processes in physics and chemistry.

[pcbi-0020147-b028] Wolynes PG, Onuchic JN, Thrumalai D (1995). Navigating the folding routes. Science.

[pcbi-0020147-b029] Wang J, Verkhivker GM (2003). Energy landscape theory, funnels, specificity and optimal criterion of biomolecular binding. Phys Rev Lett.

[pcbi-0020147-b030] Torres NV (1994). Modeling approach to control of carbohydrate metabolism during citric acid accumulation by Aspergillus niger. I. Model definition and stability of the steady state. Biotechnol Bioeng.

[pcbi-0020147-b031] Wang J, Huang B, Xia XF, Sun ZR (2006). Funneled landscape leads to robustness of cellular networks: MAPK signal transduction. Biophys J Lett.

[pcbi-0020147-b032] Kubo R, Toda M, Hashitsume N (1991). Statistical physics II.

