# Correction: Funneled Landscape Leads to Robustness of Cell Networks: Yeast Cell Cycle

**DOI:** 10.1371/annotation/b5701e25-e5f8-402f-992e-647e3f1f59b7

**Published:** 2013-09-02

**Authors:** Jin Wang, Bo Huang, Xuefeng Xia, Zhirong Sun

**Clarification to the Methods section**

The detailed description of the methods used also appears in the Supplementary Material of [31], published and subsequently retracted by Biophysical Journal.

**Correction to the Results/Discussion section**

In the subsection of the Results/Discussion section entitled ‘Conclusions’, the first sentence of the second paragraph should have referenced [31] instead of [28]. Reference [31] has now been retracted.

The correct sentence should read:

“We also worked on a different model for MAP Kinase signal transduction network [31].”

